# Pancreatic islets seeded in a novel bioscaffold forms an organoid to rescue insulin production and reverse hyperglycemia in models of type 1 diabetes

**DOI:** 10.1038/s41598-020-60947-x

**Published:** 2020-03-09

**Authors:** Diana M. Elizondo, Nailah Z. D. Brandy, Ricardo L. L. da Silva, Tatiana R. de Moura, Jamel Ali, Dazhi Yang, Michael W. Lipscomb

**Affiliations:** 1 Department of Biology, Howard University, Washington, DC United States; 2 Laboratório de Imunologia e Biologia Molecular-Hospital Universitário, Universidade Federal de Sergipe, Aracaju, Brazil; 3 Department of Chemical and Biomedical Engineering, FAMU‐FSU College of Engineering, Tallahassee, Florida 32310 USA

**Keywords:** Diseases, Endocrinology, Medical research, Molecular medicine

## Abstract

Therapeutic approaches to combat type 1 diabetes (T1D) include donor pancreas transplantation, exogenous insulin administration and immunosuppressive therapies. However, these clinical applications are limited due to insufficient tissue compatible donors, side effects of exogenous insulin administration and/or increased onset of opportunistic infections attributable to induced global immunosuppression. An alternative approach to alleviate disease states is to utilize insulin-producing pancreatic islets seeded in a bioscaffold for implantation into diabetic recipients. The present studies now report that a newly developed cationic polymer biomaterial serves as an efficient bioscaffold for delivery of donor syngeneic pancreatic islet cells to reverse hyperglycemia in murine streptozotocin induced- or non-obese diabetic mouse models of T1D. Intraperitoneal implantation of pancreatic islets seeded within the copolymer bioscaffold supports long-term cell viability, response to extracellular signaling cues and ability to produce soluble factors into the microenvironment. Elevated insulin levels were measured in recipient diabetic mice upon implantation of the islet-seeded biomaterial coupled with reduced blood glucose levels, collectively resulting in increased survival and stabilization of metabolic indices. Importantly, the implanted islet-seeded biomaterial assembled into a solid organoid substructure that reorganized the extracellular matrix compartment and recruited endothelial progenitors for neovascularization. This allowed survival of the graft long-term *in vivo* and access to the blood for monitoring glucose levels. These results highlight the novelty, simplicity and effectiveness of this biomaterial for tissue regeneration and *in vivo* restoration of organ functions.

## Introduction

Diabetic hyperglycemia is a condition that results from insufficient insulin production^1^. For type 1 diabetes (T1D), this is due to autoimmune-mediated destruction of the pancreatic islet compartment leading to deregulation of glucose-responsive insulin production from beta cells^2–4^. Though subcutaneous exogenous insulin delivery is the standard route for regulating glucose levels in diabetics, it is associated with repetitive injection pain, lipodystrophy, noncompliance and peripheral hyperinsulinemia. Furthermore, common side effects of regular insulin injections can lead to hypoglycemia, weight gain, headaches and flu-like symptoms. Therefore, novel methods to deliver insulin in minimally invasive yet clinically-effective means are needed.

Approaches to repopulate beta cell mass in diabetic patients through whole organ pancreas transplants and intrahepatic islet transplantation using donor-derived tissues have had limited success^5,6^. Failures are largely due to donor graft rejection driven by allogeneic cell mediated-immune responses and hypoxic environmental conditions within the transplant^7–12^. Therefore, engineering of a microenvironment for the transplanted islets that provides both immune tolerance and efficient vascularization within the transplant microenvironment are ideal for long-term retention and adequate glucose responsive-insulin production^13,14^.

In these studies, building on previous approaches to islet implantation^6,15–17^, an innovative new class of polysaccharide-polyamine copolymers is employed as a bioscaffold system to correct hyperglycemia in both streptozocin (STZ)-induced and autoimmune-driven non-obese diabetic (NOD) mice models^13,18^. Under physiological conditions, the protonated copolymeric scaffold biomaterial efficiently interacts with negatively charged plasma membranes of pancreatic islet cells. The surface charge interactions allow the cellulosic material to associate and aggregate with the seeded beta cells *ex vivo*, further supporting cellular infiltration into the porous substructure.

These studies have found that intraperitoneal (i.p.) implanted donor-derived syngeneic islet seeded into the biomaterial reduced hyperglycemia levels and improved metabolic hormone balances in recipient diabetic mice. Importantly, the islet-seeded copolymeric scaffold electrostatically assembled into a surrogate pancreas-like organoid that maintained the islet microenvironment, which included neovascularization within the transplant to prevent hypoxia and provide access to monitor blood glucose levels for reduction of hyperglycemia in recipients.

## Results

### Biomaterial co-polymer properties

The patented bioscaffold (or biomaterial) utilized in these studies was developed in house at Howard University. The approach included selectively oxidizing cellulose, covalently cross-linking 2,3 di-aldehyde cellulose with polyamine polymers and reducing the carbon-nitrogen double bonds of the imines. Microscopy (Supplemental Fig. 1A-C) and Fourier-transform infrared spectroscopy (FTIR; Supplemental Fig. 1D) were performed on samples to assess macroscopic and molecular structures, with FTIR noting comparison between originating cellulose structure to the final co-polymer product. Characteristic peaks at 2940-2830 cm−1 (—C—H stretching), 1576 cm−1 (—N—H bending) and 1350-1000 cm−1 (—C—N stretching) can be found in the spectrum of the novel copolymer derived from cellulose. Additionally, the spectrum of the novel cationic polymer displays a distinct peak at 1656 cm−1, which is the stretching band of —C=N, indicating the Schiff reaction between the amine groups of polyethyleneimine and the aldehyde groups of 2, 3-dialdehyde cellulose.

### Pancreatic islets seeded in the bioscaffold retain ability to produce insulin

**Figure 1:**
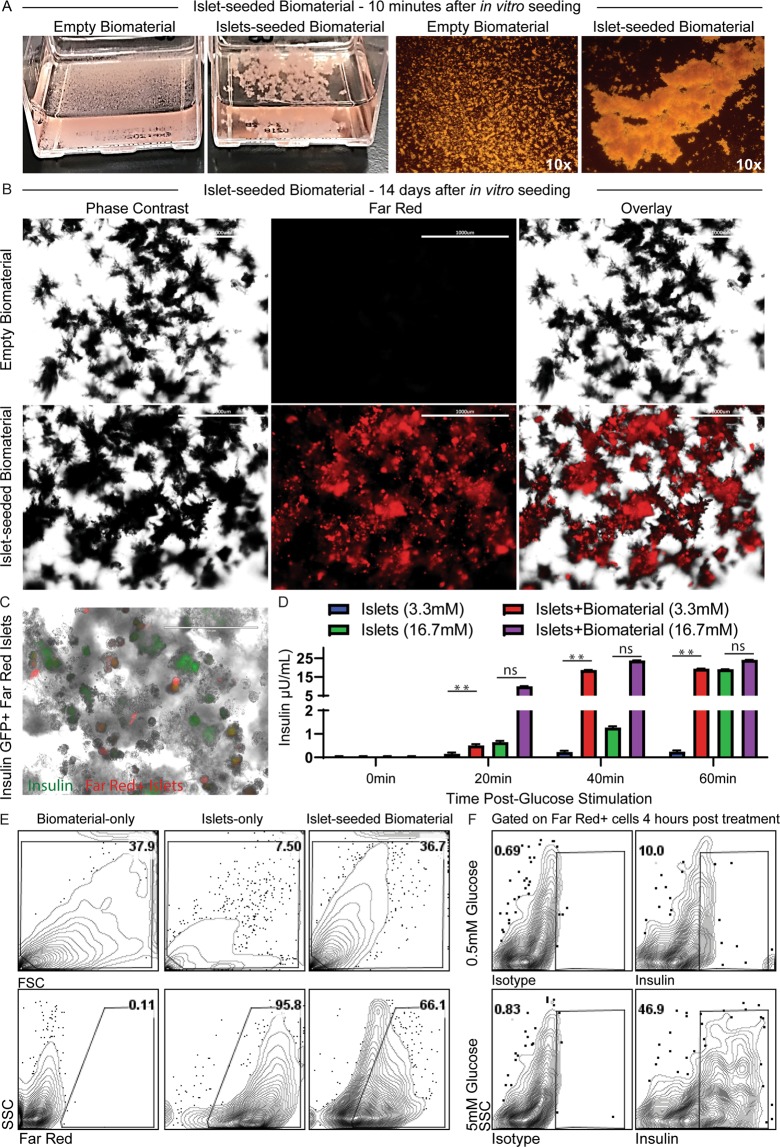
Pancreatic islets seeded in the bioscaffold produces insulin in response to glucose stimulation *in vitro*. (**A**) Images of aggregation of pancreatic islets seeded in biomaterial within 10 min after being added *in vitro*. Empty biomaterial shows original small non-aggregated fine structures. (**B**) Phase contrast and fluorescence microscopy imaging of Far Red dye-labeled islets seeded into the biomaterial after 14 days. Empty biomaterial served as control. Scale bar represents 1 mm. (**C**) Phase contrast and fluorescent imaging overlay of Far Red dye-labeled insulin-GFP^+^ islets seeded into the biomaterial. Scale bar represents 200 μm. (**D**) GSIS assays performed on islet-seeded biomaterial vs. islet-only controls. Supernatant collected at 20 min intervals over 60 min prior to assessing for insulin production by ELISA. (**E**) Flow cytometric gating strategy of Far Red-labeled islets dissociated from the biomaterial; controls include empty biomaterial (biomaterial-only) and Far red-labeled islets-only groups. (**F**) Flow cytometric analyses of intracellular insulin production from islet-seeded biomaterial under 0.5 mM-basal vs. 5.0 mM-high glucose treatment for 4 h. Gates were established based on unlabeled groups and isotype controls. For bar graphs, paired student *t* test was utilized for statistical analysis. A p value <0.05 was considered statistically significant; * is p <0.05, ** is p <0.01 and ns = not significant. Error bars indicate standard error of the mean (SEM).

The bioscaffold without seeding of any cells (empty biomaterial) is a fine structure of monomeric particles (Fig. 1A). However, addition of murine pancreatic islets to the biomaterial (islet-seeded biomaterial) results in an immediate aggregation *in vitro*. Reorganization of the scaffold assembly occurred within 3-4 h after initial seeding (Supplemental Fig. 1E), with complete embedding of the cells into the biomaterial within 2 weeks of *in vitro* co-culture (Supplemental Fig. 1F). Scanning electron microscopy performed on empty biomaterial at 10,000x magnification and islet-seeded biomaterial at 3,500x magnifications (Supplemental Fig. 1G-H) highlights the interactive aggregation between the pancreatic islet cells and cellulosic co-polymer biomaterial. To further characterize islets seeded within the biomaterial, harvested pancreas islets were labeled with Far Red dye *ex vivo* just prior to seeding in the biomaterial. After 14 days of *in vitro* culturing, phase contrast overlay with fluorescence microscopy shows Far Red-labeled islets stably aggregated with the biomaterial (Fig. 1B). Using islets harvested from mice carrying green fluorescent protein (GFP) under the insulin promoter, fluorescence microscopy overlay of Far Red-labeled islets show that the seeded cells in the biomaterial were able to drive the insulin promoter to produce GFP (Fig. 1C). For visualization, the intensity of the Far Red fluorescence channel was reduced substantially to show the dim GFP expression from insulin-producing beta cells within the islet-seeded biomaterial.

Functionality of the islets seeded within the biomaterial was assessed by measuring insulin responses upon *in vitro* glucose stimulation. Briefly, islets seeded in the biomaterial were incubated in basal media (3.3 mM glucose) prior to stimulation with high glucose solution (16.7 mM glucose). Supernatant was then harvested every 20 min for 1 h. Islets without seeding in the biomaterial (islets-only) served as internal control. Results revealed that the islets seeded within the biomaterial were able to sufficiently produce insulin in response to glucose, surprisingly more robust to that of the islets-only group (Fig. 1D). These results establish that insulin can be secreted and released out of the biomaterial into the supernatant; molecules are not trapped within the biomaterial scaffold network. As a corroborative index, Far Red-labeled islets seeded in the biomaterial were stimulated with glucose prior to freeing the labeled cells from the bioscaffold by gentle disassociation. These pancreatic islet cells were then assessed by flow cytometric analyses for insulin expression 4 h after glucose stimulation (Fig. 1E,F). Results revealed that the islet-seeded biomaterial had an increase in frequency of insulin^+^ pancreatic cells from 10.0% ± 2.4 to 46.9% ± 6.7 upon addition of 5 mM glucose.

### Implantation of islet-seeded biomaterial reduces hyperglycemia in diabetic mice

**Figure 2:**
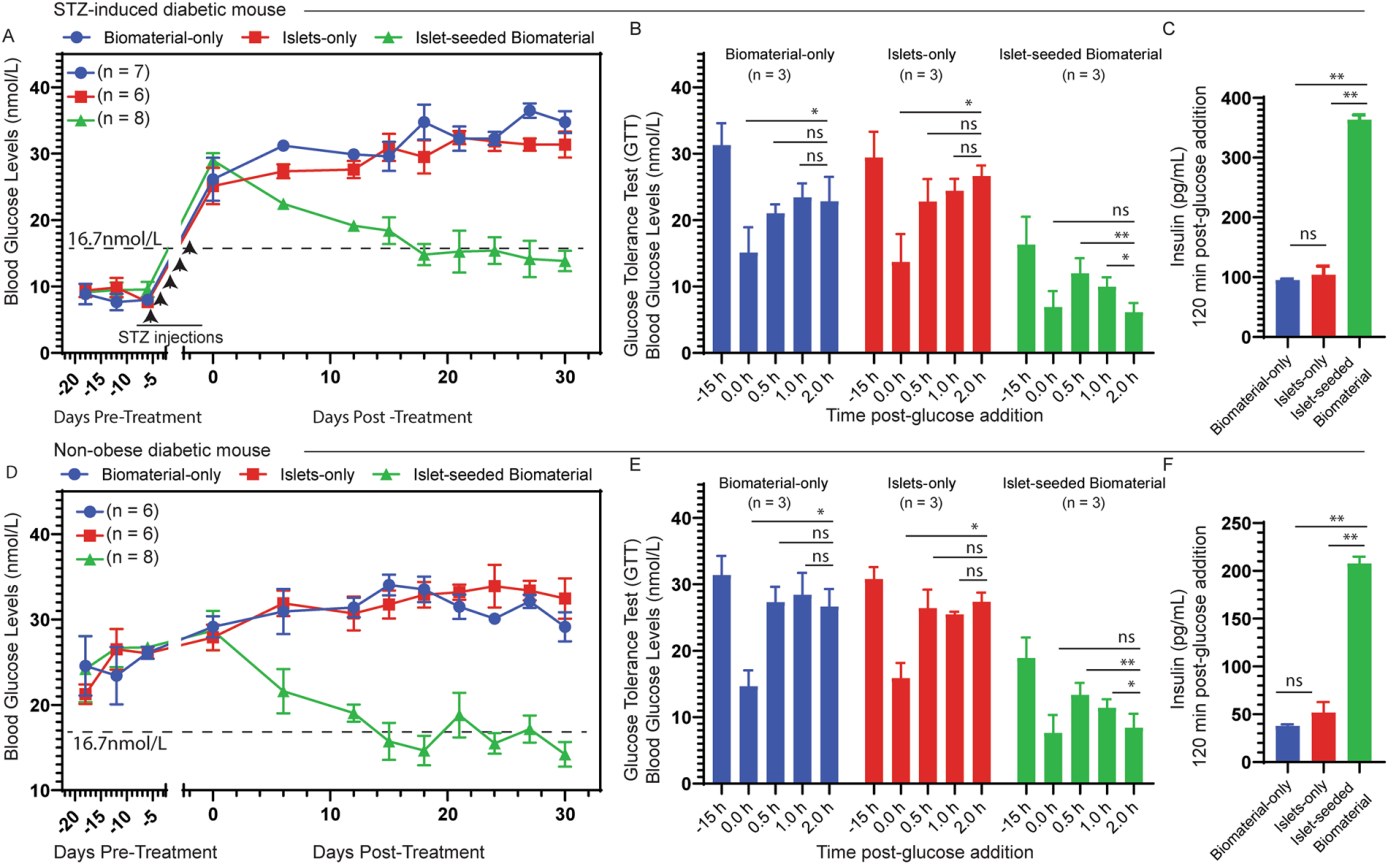
Implanted islet-seeded biomaterial into diabetic mice reduces glucose levels. Islet-seeded biomaterial or respective controls were injected intraperitoneal into both STZ-induced and NOD diabetic mice. STZ-induced diabetic mice were treated with biomaterial-only (n = 7), islets-only (n = 6) or islet-seeded biomaterial (n = 8). Days −25 through 0 designate pre-treatment period to identify baseline glucose levels. STZ was injected day −7 through day −3 (for a total of 5 days) to induce diabetes. (**A**) STZ-induced diabetic mice assessment of blood glucose levels recorded over the treatment time period. Dashed horizontal line represents 16.7 nmol/L marker for diabetes determination. (**B**) Glucose tolerance test (GTT) performed in STZ-induced diabetic mice on day 20 post-implantation treatment with islets-only, biomaterial-only or islet-seeded biomaterial. (**C**) Total blood insulin levels were measured 120 min after GTT performed in STZ-induced diabetic mice 20 days post-implantation treatment. (**D**) NOD diabetic groups were treated with biomaterial-only (n = 6), islet-only (n = 6) or islet-seeded biomaterial (n = 8). Blood glucose levels recorded over 30 days post-implantation. (**E**) GTT performed in NOD treated mice on day 20 post-implantation. (**F**) Total blood insulin levels measured after GTT performed in NOD treated groups on day 20 post-implantation. Paired student *t* test was utilized for statistical analysis. A p value < 0.05 was considered statistically significant; * is p < 0.05, ** is p <0.01 and ns = not significant. Error bars for all figures indicate standard errors of the mean (SEM).

After corroboration of islet cells’ functionality upon *in vitro* seeding into the biomaterial, studies next evaluated their ability to reduce glucose levels and increase survival of diabetic mice *in vivo*. Islet-seeded biomaterial, islets (islets-only) or empty biomaterial (biomaterial-only) was i.p. injected into STZ induced-diabetic mice with glucose levels in the range of 26.8 ± 3.1 mmol/L. The STZ model was employed to exclude beta cell autoantigen-driven autoimmunity as a variable. Reduced glucose levels in the cohort that received the islet-seeded biomaterial treatment were observed (Fig. 2A), whereas glucose levels continued to elevate in control recipients receiving implants of islets-only or biomaterial-only controls. Glucose tolerance tests (GTT) were performed by i.p. injection 3 weeks after implantation of islet-seeded biomaterial or controls into recipient diabetic mice. Results revealed decreased blood glucose levels in the islet-seeded biomaterial treated mice, but not controls (Fig. 2B). Lastly, insulin levels were markedly higher in the islet-seeded biomaterial treatment groups compared to controls at the end of the GTT assay (Fig. 2C). Next, using the non-obese diabetic (NOD) mouse model, analogous studies were performed under autoimmune settings. Results of islet-seeded biomaterial implantation recapitulated that of the STZ-induced diabetic model, whereby there was lowered glucose levels (Fig. 2D), decreased blood glucose upon GTT (Fig. 2E) and increased total insulin levels (Fig. 2F) in the islet-seeded biomaterial implantation group, but not controls. It is important to note that implantation with islet-seeded biomaterial in the STZ-induced diabetic mice resulted in long-term survival of the mice, with 4 out of 7 treated animals surviving beyond 120 days post-implantation (Supplemental Fig. 2A). Islets-only or biomaterial-only treated groups died of severe morbidity ~60 days after implantation. However, within the NOD mouse model, studies revealed that survival of mice treated with islet-seeded biomaterial was prolonged by 2.5-fold over control groups (Supplemental Fig. 2B). This may suggest that autoreactive immune cell infiltration may be disrupting the function of the i.p. injected islet-seeded biomaterial after extended periods within the NOD diabetic recipient mice.

### Partial rescue of metabolic levels upon treatment with pancreatic islet-seeded bioscaffold

**Figure 3:**
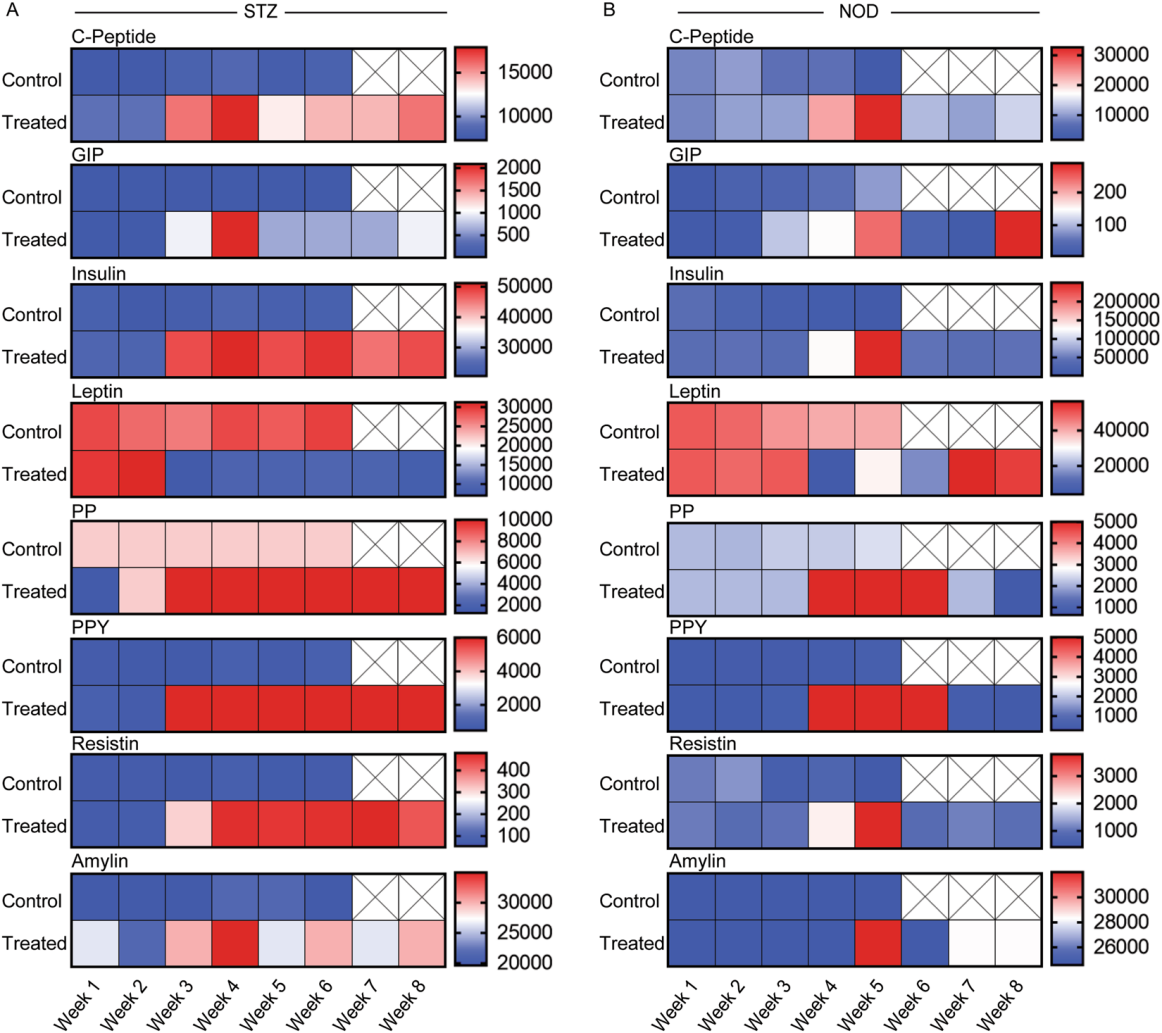
Partial rescue of metabolic indices upon islet-seeded biomaterial implantation into diabetic mice. Islet-seeded biomaterial (n = 6) was intraperitoneal injected into both STZ and NOD mice; control group is empty biomaterial (biomaterial-only; n = 6). Serum samples were collected weekly and assessed for C-peptide, GIP, insulin, leptin, PP, PPY, resistin and amylin hormone secretion in recipient (**A**) STZ-induced and (**B**) NOD diabetic mice. Experiments were performed in replicates with 6 mice per group. Data is presented as mean. No values determined after week 6 for STZ-induced and week 5 for NOD diabetic mice due to increasing states of severe morbidity in the control groups.

After implantation, blood serum was collected weekly for 8 weeks. Measures of metabolic indices were evaluated comparing treated islet-seeded biomaterial vs. empty biomaterial (biomaterial-only) controls in the STZ-induced diabetic mice. Results showed increased C-peptide, GIP, insulin, PP, PPY and resistin levels, with leptin levels significantly lowered, in diabetic mice receiving the islet-seeded biomaterial (Fig. 3A). Analogous results were observed in the NOD model, where metabolic levels began to stabilize for 3–6 weeks post islet-seeded biomaterial implantation (Fig. 3B). However, metabolic levels began to destabilize after the 6-week mark, as seen with lowered C-Peptide, GIP, insulin and PP. Therefore, in comparison with STZ-induced diabetic, islet-seeded biomaterial began to fail under autoimmune settings in the NOD diabetic model, which corresponds with observed reduced long-term survival.

### Islet-seeded biomaterial drives neovascularization for long-term function and viability

**Figure 4:**
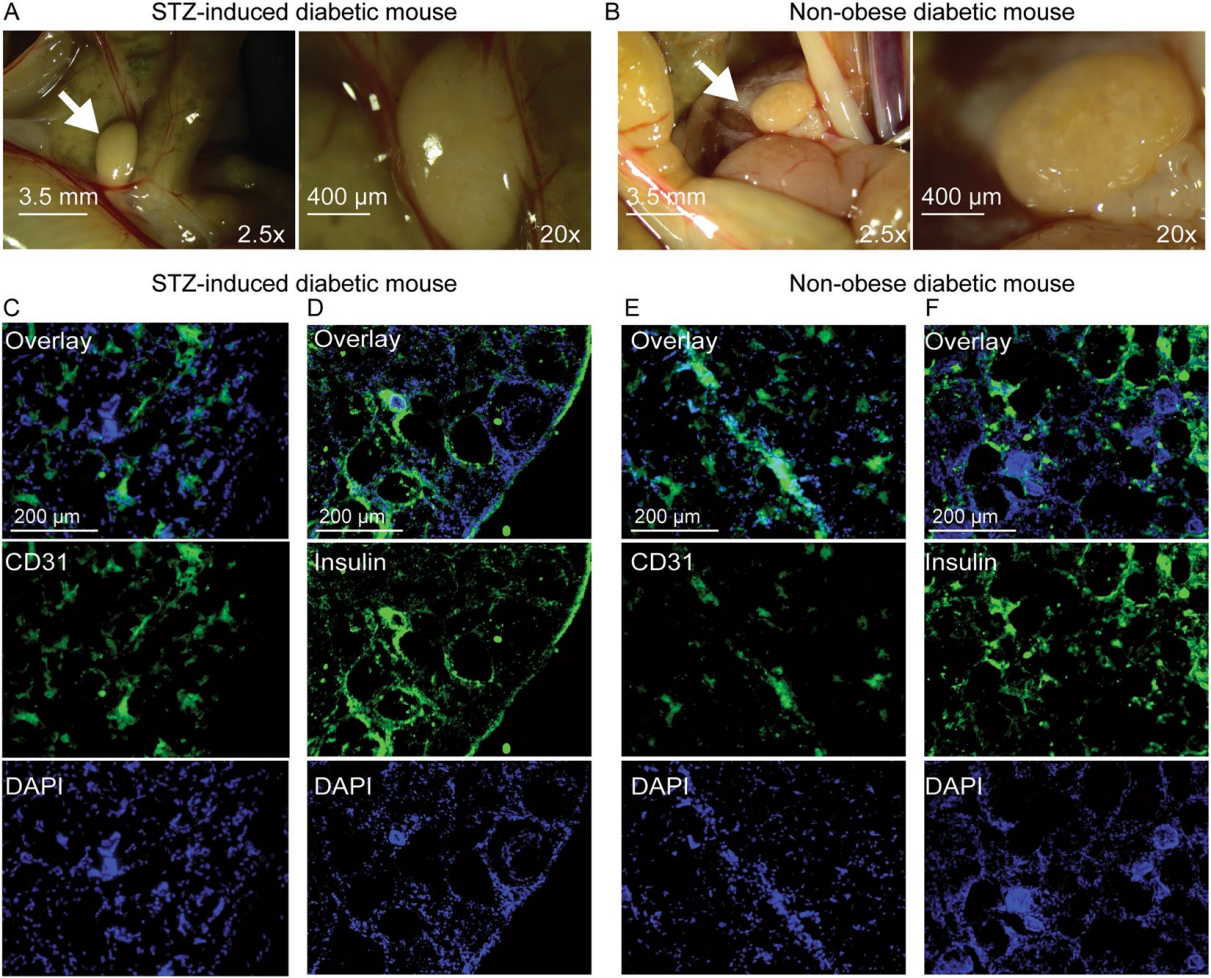
Islet-seeded biomaterial forms an organ-like structure *in vivo* that promotes neovascularization. Stereomicroscope imaging of resected islet-seeded biomaterial 5 weeks after initial i.p. implantation into (**A**) STZ-induced and (**B**) NOD mice peritoneal cavity at 2.5x and 20x magnifications. Arrow indicates location of the aggregated islet-seeded biomaterial substructure nearest small intestines and (original site of) pancreas. Fluorescence microscopy images of excised and sectioned islet-seeded biomaterial from STZ-induced diabetic mice stained for (**C**) CD31 and (**D**) insulin and from NOD mice stained for (**E**) CD31 and (**F**) insulin; DAPI used as nuclear staining dye.

Implanted biomaterial seeded with islets was resected from sacrificed STZ-induced diabetic and NOD treated mice 5 weeks after i.p. implantation. Analyses revealed that the islet-seeded biomaterial aggregated around the intestinal area closest to the pancreas (Fig. 4A,B); arrows show the *in vivo* self-assembled islet-seeded biomaterial as an organ-like structure approximately 3 mm in diameter within implanted STZ-induced and NOD diabetic recipient mice. Importantly, this was only observed in islet-seeded biomaterial treated groups, as no aggregated assembly was found in implanted biomaterial-only groups. No islet-seeded biomaterial was observed in or near the lungs, liver, heart or brain (data not shown). Cryosections of resected islet-seeded biomaterial were prepared prior to staining for insulin or the endothelial cell marker CD31. In the STZ-induced diabetic model, both insulin and CD31 expression are present, confirming results of long-term insulin production and vascularization for supporting implant survival and access to monitoring blood glucose levels (Fig. 4C,D). Harvested islet-seeded biomaterial in STZ-induced diabetic treated mice 10 weeks after initial implantation continued to show the solid organ-like structure containing islets supported by vascularization (Supplemental Fig. 3). Similar profiles of endothelial marker recruitment were observed in the NOD diabetic groups that received islet-seeded biomaterial (Fig. 4E). However, lower overall levels of insulin-producing cells within the implanted islet-seeded biomaterial were observed (Fig. 4F).

### Implantation of islet-seeded biomaterial does not elicit inflammatory responses

**Figure 5:**
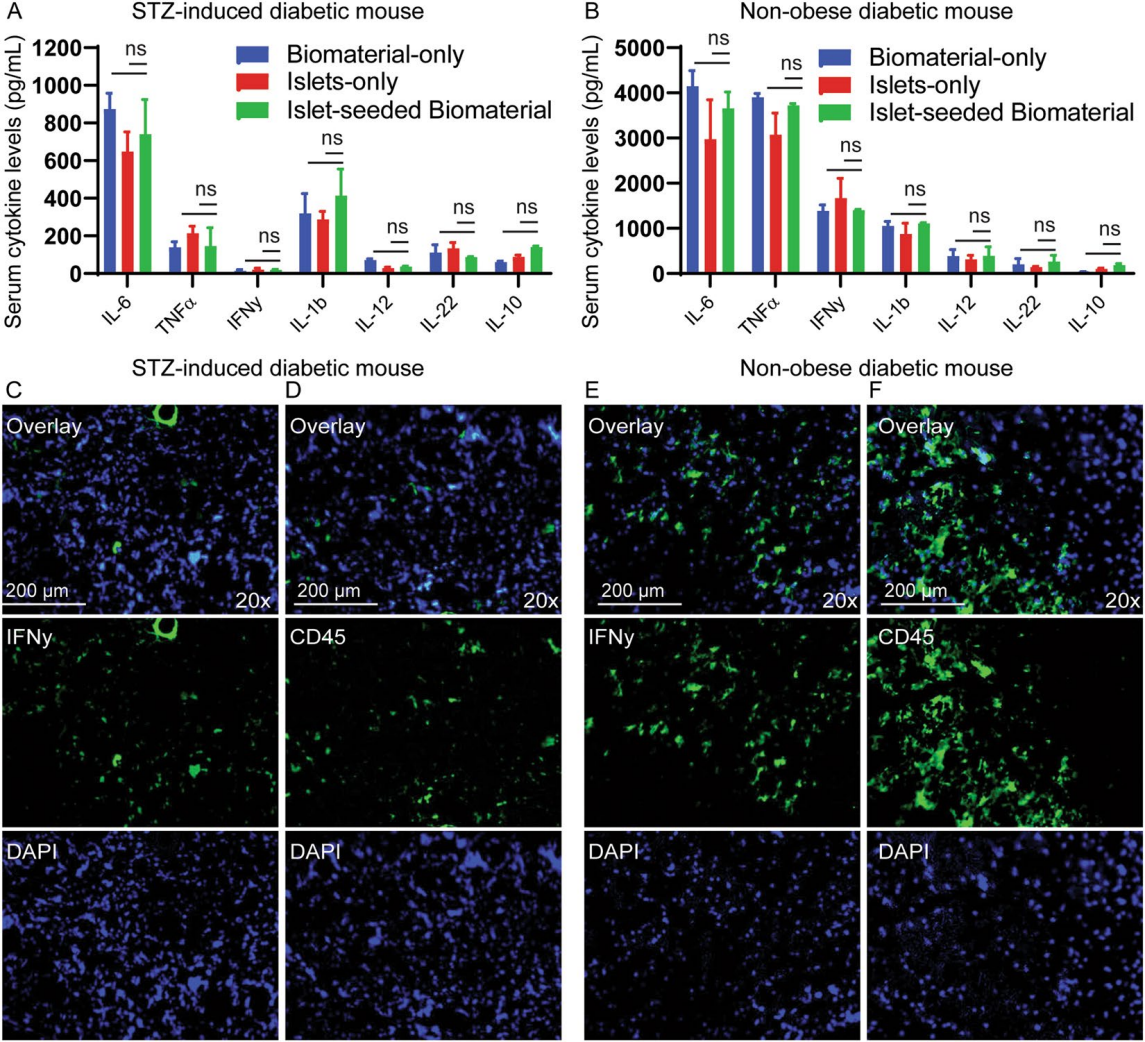
No significant changes in inflammation upon islet-seeded biomaterial implantation. Luminex panel analyses on collected serum for IL-6, TNFα, IFNγ, IL-1β, IL-12, IL-22 and IL-10 inflammatory cytokines from (**A**) STZ-induced and (**B**) NOD diabetic mice after treatment with islet-seeded biomaterial (n = 6); control is empty biomaterial-only (n = 6). Experiments were performed in replicates with 6 mice per experimental group. Data is presented as mean ± SEM. Paired student* t* test was utilized for statistical analysis. A p value < 0.05 was considered statistically significant; * is p <0.05, ** is p < 0.01 and ns = not significant. Error bars for all figures indicate standard errors of the mean (SEM). Excised islet-seeded biomaterial was sectioned and stained with (**C**,**E**) IFNγ or (**D**,**F**) CD45 after 20 days post-implantation in STZ-induced and NOD diabetic mice; DAPI served as nuclear staining dye.

Serum from islet-seeded biomaterial treated diabetic mice was collected 5 weeks after implantation and assessed for inflammatory cytokines. Results showed no significant differences in IL-6, TNFα, INFγ, IL-1β, IL-12, IL-22 or IL-10 between the islet-seeded biomaterial vs. islet-only and biomaterial-only controls in STZ-induced or NOD diabetic recipients, suggesting no systemic inflammation caused by implantation of the biomaterial (Fig. 5A,B). As expected, elevated levels of pro-inflammatory cytokines were detected in the NOD model. There was no observable direct reversal of autoimmune events in the treated NOD diabetic model, as measured by serum cytokine levels, suggesting that implantation of islet-seeded biomaterial does not abrogate autoimmunity. In order to investigate infiltration of immune cells, excised biomaterial was harvested and sectioned from implanted mice. Fluorescence microscopy analyses showed no infiltration of CD45^+^ leukocytes or IFNγ-producing cells into the islet-seeded biomaterial within the STZ-induced diabetic mouse (Fig. 5C,D). However, for the NOD model, results showed infiltration of CD45^+^ immune cells and presence of IFNγ in the resected islet-seeded biomaterial (Fig. 5E,F). Monoclonal anti-CD3 neutralization treatment markedly reduced, but did not abolish, immune cell infiltration and re-emergence of diabetic pathologies in the NOD cohorts (data not shown). Taken together, the islet-seeded biomaterial can reduce hyperglycemia by restoring insulin levels in a glucose-responsive manner in both STZ-induced and autoimmune-driven NOD diabetic mouse models. Success of the clinically relevant approach shows efficacy in establishing long-term retention as a surrogate-like organ that is able to effectively induce neovascularization for access to oxygen, nutrients and monitoring of blood glucose levels.

## Discussion

These studies show the effectiveness of this new cationic copolymer biomaterial in serving as an effective bioscaffold for seeding pancreatic islet cells and formation of a self-sufficient organoid to reduce hyperglycemia upon implantation in diabetic recipients. Cells can grow on both the exterior and interior surfaces of the cationic charged surface of the biomaterial, which better mimics 3D growth patterns. This unique capability also supports nutrient infiltration during anchorage-dependent cell growth and viability. The result of the observed islet-biomaterial assembly is a densely interlocked network that well supports cellular functions and interactions (i.e. infiltration, reorganization, signaling and secretion) under physiological conditions. While conventional microcarriers are often used for cultivating cells in a suspended state by supporting cell proliferation in a single layer on the surface, the cationic copolymer biomaterial used in these studies advantageously provides multiple layers with porous structures. The size of the bioscaffold, as well as the pore size, can be controlled by the reaction conditions to optimally support a variety of cell types. In this respect, simple chemical modifications and sheering approaches provide an advantageous means to produce the biomaterial with uniform size and variable sized pores, allowing generation of median particle sizes ranging from 100 μm to about 1 mm and pores between 30 and 300 μm. Thus, the system allows a high cell-to-carrier ratio for cell seeding and culturing.

Seeding the pancreatic islets within the biomaterial allows for maximal occupancy, with the cationic copolymer serving as a scaffold for interacting strongly with the charged surface of cells. This is an important innovation in design, as surfaces of many existing microcarriers generally need to be treated (e.g., by an attachment protein) to enhance the anchorage/affinity of cultured cells^19,20^. However, the inherent electrostatic state of the bioscaffold assembly allows immediate and long-term retention of pancreatic islet cells, as well as subtle reorganization of the copolymer around the cells to create a stable niche and organoid substructure.

Visualization of the islet-seeded biomaterial *in vitro* revealed viable insulin-producing pancreatic cells by fluorescence microscopy, as observed by expression of GFP driven under the insulin promoter. Notably, fluorescence intensity levels for insulin were variable. This may be attributed to the different levels of insulin based on various stages of cell activity and/or maturation under steady-state conditions^21^. Furthermore, due to the intrinsic brightness of the Far Red dye signal relative to the lowly expressed GFP fluorescence intensity, levels of Far Red were greatly reduced during imaging and post-acquisition analyses to compensate for the dimly expressed GFP^+^ cells.

Beta cells within the islet-seeded biomaterial were able to both produce and secrete insulin into the supernatant in direct response to glucose, as detected by flow cytometric analyses and ELISA. Interrogating cells by flow cytometric analyses confirmed both cell viability and intracellular production of insulin, with levels increasing in direct response to glucose addition to the culture. Collection of supernatant after varying time points proved that the scaffold biomaterial supports release of molecules into the extracellular spaces, while providing a stable aggregate niche for the seeded cells to stay in place. Taken together, the pancreatic islet cells seeded into the biomaterial remained viable for 4 weeks *in vitro* and small molecules can diffuse passively in-and-out of the bioscaffold.

Implantation of islet-seeded biomaterial into either STZ-induced or NOD diabetic mice resulted in significant reduction of hyperglycemia coupled with marked increase in insulin levels. Both treated STZ-induced and NOD diabetic mice lived longer than control littermates. However, reduced pathology in the NOD group was limited due to subsequent autoimmune cell infiltration into the islet-seeded biomaterial implant. Thus, as expected, the islet-seeded biomaterial does not directly alleviate immune responses; infiltration of immune cells was detected upon resection of the islet-seeded biomaterial from the treated animals. Therefore, combinatorial treatments of islet-seeded biomaterial with antigen-specific immunosuppressive agents, such as dendritic cell-based vaccines to silence autoreactive T cells, would best serve to complement this insulin restorative strategy. Future studies are currently underway addressing this approach by utilizing tolerogenic dendritic cells and *ex vivo* expanded regulatory T cells co-seeded within the biomaterial along with the pancreatic islets.

Glucose and metabolic levels were stabilized consistently 2-3 weeks after implantation of islet-seeded biomaterial in both STZ-induced and NOD diabetic recipients. This included increased levels of insulin, C-peptide, GIP, PP PPY and amylin in islet-seeded biomaterial treated groups, but not empty biomaterial or islet-only controls. However, results also showed moderate increases of resistin, associated with obesity and type 2 diabetes in islet-seeded biomaterial treated diabetic mice^22^. It is possible that the sudden rapid production of insulin from the implanted islet-seeded biomaterial triggers an early negative-feedback mechanism to drive insulin resistance. Furthermore, lowered leptin levels found in the treated mice suggest decreased insulin sensitivity and impaired ability to suppress appetite^23,24^. Although there was no noticeable difference in appetite of mice during weekly post-implantation monitoring, changes in leptin could be a result of the dramatic lowering of hyperglycemia.

Upon intraperitoneal implantation of the islet-seeded biomaterial, self-aggregation into a larger macromolecular substructure resembling an organ-like structure was identified. The diameter of the explanted islet-seeded biomaterial ranged between 3 and 4 mm, which is about half the typical 5–8 mm length of the adult mouse heart and twice as large as a lymph node. An important component of long-term retention *in vivo* of adoptively transferred cells is access to the vasculature. Strikingly, the islet-seeded biomaterial induced neovascularization events to form branched access to neighboring blood vessels within the self-assembled structure. The studies took care to deplete endothelial cells from *ex vivo* isolated pancreatic islets using antibodies to deplete CD31^+^ and VEGFR2^+^ subsets by magnetic bead isolation approaches prior to seeding and i.p. implantation in recipient diabetic mice. This prevented potential endothelial cells from being carried over upon *in vivo* transfer.

It remains to be determined how the seeded islets in the sheered biomaterial survived for the first weeks of implantation prior to aggregation and neovascularization events. Previous reports have shown that isolated islets can survive *in vivo* if oxygen supply is available through diffusion from the surrounding well-oxygenated tissue after transplantation^25^. Furthermore, hypoxic conditions of the islet-seeded biomaterial implanted into the peritoneal space may drive recruitment of endothelial progenitors to promote angiogenesis. Additionally, glucose and inflammation can directly control vascularization, suggesting that the implantation coupled with high levels of glucose can further support recruitment of endothelial cells to form new vasculature^26^. It is also possible that macrophages infiltrated into the islet-seeded biomaterial to help drive vascular endothelial growth factor-driven angiogenesis^27,28^.

Overall, these studies demonstrate that this new class of copolymer biomaterials seeded with islets can support cell viability and glucose-responsive insulin production to restrain hyperglycemia in diabetic recipients. Importantly, upon implantation, the pancreatic islets seeded in the bioscaffold form an organ-like structure that directly promotes long-term sustainability by inducing neovascularization. The use of this copolymer biomaterial would directly address key requirements for recapitulating an islet microenvironment supporting long-term insulin production in a physiological-relevant manner to elevated blood glucose. These features highlight the feasibility for effective insulin restoration and opens the door to additional applications to rescuing other types of organ failures using this new class of biomaterials.

## Methods

### Biomaterial assembly

For biomaterial preparation, the patented approach includes selectively oxidizing cellulose, covalently cross-linking 2,3 di-aldehyde cellulose with polyamine polymers and reducing the carbon–nitrogen double bonds of the imines. Importantly, 2,3 di-aldehyde cellulose covalently cross-links with the functional block polymers to form the polyamine cellulosic copolymers with a three-dimensional densely interlocked network. Amine groups were protonated under an aqueous environment with a pH lower than 9 allowing the positively charged copolymeric scaffold to form hydrogel matrices. Functional group characterization of the material was evaluated using Fourier Transform Infrared Spectroscopy (FTIR) analysis. Compete details of the patented biomaterial is available through patent US 20180094080 (April 2018).

### FTIR spectra sample preparation and analysis

Fourier transform infrared spectroscopy was performed to determine the chemical structure of the cationic copolymer biomaterial prior to cell seeding. For infrared analysis, synthesized biomaterials were lyophilized to obtain powders. Spectra of these samples falling in the range between 400–4000 cm-1 were obtained using a Thermo Scientific Nicolet 6700 FT-IR spectrometer.

### SEM sample preparation and analysis

The biomaterial was co-cultured in fetal bovine serum (FBS) supplemented RPMI medium with (islet-seeded biomaterial) or without islets control (empty biomaterial). Islet-seeded biomaterial was incubated for 24 h at 37 °C *in vitro*. Next, aggregated pieces of islet-seeded biomaterial or empty biomaterial were transferred into 2% buffered glutaraldehyde. Samples were fixed overnight at 4 °C. After fixation, samples were rinsed with 0.1 M HEPES buffer 3x for 5 min each with gentle agitation followed by extensive rinsing. Preparations were dehydrated using an alcohol serial dehydration approach. Next, samples were chemically dried through incubation in gradients of HMDS for 15 min each prior to placing onto clean silicon chips and sputter coated with a thin film (~15 nm) of palladium. Ultrastructure images were acquired via FEI Helios G4 field emission scanning electron microscope operated at an acceleration voltage of 3 kV.

### Mice

Both male and female C57BL/6 (wild type; WT), GFP reporter under the insulin promoter on C57BL/6 background (INS-GFP), and NOD.CB17-Prkdc^scid^ (NOD.SCID) mice at 8–12 weeks of age were used as a source for pancreatic islets. NOD/ShiLtj (non-obese diabetic; NOD)^29,30^ and NOD.CB17-Prkdc^scid^ (NOD.SCID) female mice at 16–20 weeks of age served as recipients of intraperitoneal (i.p) implantations with islet-seeded biomaterial, islets-only or biomaterial-only. NOD diabetic mice received syngeneic pancreatic islets derived from NOD.CB17-Prkdc^scid^ donor mice. For streptozotocin (STZ)-induced diabetes, low dose STZ (Cayman Chemicals; Ann Arbor, MI) (12 g/kg) treatment was i.p. injected each day over the course of five days into both male and female C57BL/6 WT mice at ages 10–16 weeks old^31,32^. STZ-induced diabetic mice received syngeneic pancreatic islets from healthy (i.e. non-STZ treated) donor WT mice. Littermates were used for age- and sex-matching. Animal procedures were performed in accordance and approved by the Institutional Animal Care and Use Committee. Mice were purchased from The Jackson Laboratory (Bar Harbor, ME) and housed in pathogen-free facilities at Howard University.

### Glucose monitoring

Glucose monitoring using the Aviva Accu-Check (Roche; Indianapolis, IN) glucometer by tail vein prick was performed twice a week to assess onset and progression of diabetes in both STZ-induced and NOD mice. Tails of mice were pricked to collect 2 µl of blood for immediate reading on glucometer test strips. Diabetes was identified as values > 16.7 mmol/L of non-fasted mice. Hyperglycemic states identified as values 11–16.6 nmol/L.

### Pancreatic islet isolation

Sacrificed donor mice were dissected using a 40X wide-field stereomicroscope for pancreas islet isolation. Islet purification steps were performed using a modified protocol from Stull *et al*.^33^. Briefly, pancreas was inflated with 200 µl of collagenase type IV at a concentration of 0.5 mg/mL in HBSS prior to injection through the hepatic vein using a 27-gauge needle. The pancreas was digested with collagenase type IV (Thermo Fisher Scientific; Waltham, MA) for 30 min at 37 °C. Next, suspension was mixed gently prior to centrifugation for 1 min. The pellet was re-suspended in HBSS and overlaid with pre-mixed histopaque 1100 and histopaque 1077 (Sigma Aldrich; St. Louis, MO) prior to centrifugation for 20 min at 330xg. The middle white layer containing islets was carefully collected and transferred to a new tube containing RPMI media. In some studies, CD31^+^ and VEGFR^+^ endothelial cells were depleted using magnetic beads; primary antibodies purchased from BioLegend and secondary antibodies conjugated to magnetic microbeads from Qiagen (Hilden, Germany). Islets were allowed to recover by incubating at 37 °C for 24 h. Next, ~2000 islets were hand-picked using a stereomicroscope and seeded into the biomaterial.

### Islet seeding into biomaterial

Biomaterial was disinfected in 70% ethanol and then washed extensively with deionized water. After washing, the biomaterial was cultured with isolated pancreatic islets in RPMI media supplemented with FBS and incubated at 37 °C.

### Dissociation of cells from the islet-seeded biomaterial

Islet-seeded biomaterial was centrifuged down prior to resuspension in collagenase type IV. The mixture was incubated on a rotating platform at 37 °C for 1 h. Next, cells were vortexed and strained using a 40 µm cell strainer. Dissociated cells were prepared for flow cytometry analyses and microscopy.

### *In vitro* glucose-stimulated insulin secretion (GSIS) assay

Stock Kreb’s buffer (KRB) insulin-stimulating media was prepared with NaCl (8.0 g/L), KCL (0.44 g/L), KH_2_PO_4_(0.16 g/L), MgSO_4_−7H_2_O(0.3 g/L), CaCl_2_−2H_2_O(0.37 g/L), NaHCO_3_(2.1 g/L). 3.3 mM glucose and 16.7 mM glucose were added to KRB to prepare basal media and glucose working solutions, respectively^34^. Islet-seeded biomaterial was transferred to basal media and incubated for 30 min at 37 °C. Islets alone (islets-only) served as internal controls. Next, glucose working solution was added to the cells for 60 min prior to incubation at 37 °C. Supernatant was harvested every 20 min (through the 60 min incubation window) to assess insulin production by ELISA (Thermo Fisher Scientific; Waltham, MA). For flow cytometric analyses of intracellular insulin production, islet cells were immediately dissociated out of biomaterial post-high glucose exposure utilizing collagenase type IV digestion (0.5 mg/mL; Thermo Fisher Scientific), fixed, stained and acquired on the BD Accuri C6 flow cytometric analyzer (BD Biosciences).

### Islet-seeded biomaterial injection into mice

After the two-week *in vitro* culture, islet-seeded biomaterial was sheered 5 times by passing through a 27-gauge needle 24 h prior to implantation. The amount of islet-seeded biomaterial used for injection per mouse held approximately 500 islets. The sheered islet-seeded biomaterial was washed extensively in PBS to remove residual media and serum prior to re-suspension to 200 µl with PBS for intraperitoneal injection into STZ-induced or NOD diabetic recipient mice. Injection of empty sheered biomaterial (biomaterial-only) and free islets (islets-only) served as controls.

### Glucose tolerance test (GTT) and collection of blood sera for metabolic indices measurement

Mice were fasted for 12 h prior to i.p. injection with dextrose (2 g/kg) bolus. Blood glucose levels were measured using a glucometer at 0, 30, 60 and 120 min. Additional blood sera was collected 120 min after dextrose injection and utilized for determining insulin levels by ELISA.

### Dyes and Luminex multiplex analyses

Far Red dye (Thermo Fisher Scientific) was used to label islets prior to seeding in the biomaterial. The dye allows for visualization of islets in the biomaterial for several days *in vitro*. For multiplex analyses, blood was collected from mice weejkt after treatment. Blood was processed into serum prior to adding to a mixture of color-coded beads pre-coated with analyte-specific capture antibodies. Biotinylated detection antibodies specific to the analytes of interest were added to form the antibody-antigen sandwich prior to reading on the Luminex MAGPIX Analyzer (Luminex; Austin TX).

### Antibodies, cryosections and fluorescence microscopy

Excised islet-seeded biomaterial from implanted mice were fixed in 3% PFA prior to transfer into a 30% sucrose solution. Sections were prepared at 10 or 20 µm thickness using a standard automated cryosectioner (Cryostat NX70; Thermo Fisher). Preparations were transferred onto poly-L-lysine coated glass slides. Samples were then permeabilized using 0.3% Triton-X solution and blocked with 0.2% BSA in permeabilization buffer. Sections were stained with insulin (cat# MAB1417; R&D Systems), CD31 (clone MEC 13.3), IFNγ (clone XMG1.2) and/or CD45 (clone 30-F11), followed by extensive washing. CD31, IFNγ and CD45 antibodies were purchased from Biolegend. Isotype control antibodies were used as internal controls. Sections were co-stained with DAPI (Thermo Fisher) nuclear staining dye prior to mounting with cover slips. Slides were imaged using the Olympus FSX100 (Olympus, Waltham MA) or Evos FL (Thermo Fisher) fluorescence microscopes.

### Hematoxylin eosin (H&E) staining

Samples were H&E stained prior to observation by phase contrast microscopy. Hematoxylin solution was used to stain samples for 10 min prior to washing with water and alcohol. Counterstain was with eosin-phloxine solution for 1 min. Standard phase-contrast images were taken using the Evos FL Manual (Thermo Scientific) inverted fluorescence microscope. Images were randomly taken from different regions within the material and the diameter of particles were measured using a CellProfiler.

### Flow cytometry

Flow cytometry approaches were performed as previously described^35–37^. Cell surface staining was performed with PBS supplemented with 1 mM EDTA and 2.5% bovine serum (FACS buffer). Cells were washed with FACS buffer prior to extracellular staining with fluorochrome-tagged antibodies. Dilutions were antibody specific, but roughly 10 µl of a 10 µg/mL working concentration was utilized per 2 ×10^5^ cells. Respective isotype controls were used in all assays. Cells were then fixed with 3% paraformaldehyde (PFA) in PBS. For intracellular antibody labeling, fixed cells were permeabilized with 0.2% saponin in PBS. Next, primary antibodies or isotype controls were added at approximately 10 µg/mL concentrations followed by washing and subsequent staining with secondary fluorochrome-labeled antibodies. Cells were acquired on a BD FACSVerse or Accuri C6 flow cytometric analyzer (BD Biosciences, San Jose CA). Datasets were analyzed using Flow Jo v10 (Flow Jo LLC; Ashland OR).

### Statistical analysis

GraphPad Prism v8.0 (GraphPad Software, La Jolla CA) was used to determine statistical significance and generates graphs and plots. Statistical analyses were performed as previously described^38^. Student unpaired two-tailed t test was used to evaluate the significance of two groups. A p value < 0.05 was considered statistically significant; * is p < 0.05, ** is p < 0.01 and *** is p < 0.001. ns = not significant. Error bars for all figures indicate standard errors. Survival data was plotted using the Kaplan-Meier method. Significance of differences between groups were tested by comparing group means and medians (mean survival time [MST]) by either the two-tailed t test or Wilcoxon’s signed-rank test.

## Supplementary information

Supplementary Data.

Supplementary Data2.

Supplementary Data3.

Supplementary Data4.
