# Supplementary material for: Pancreatic islets seeded in a novel bioscaffold forms an organoid to rescue insulin production and reverse hyperglycemia in models of type 1 diabetes
Source: Sci Rep. 2020 Mar 9;10:4362. doi: 10.1038/s41598-020-60947-x (PMC7062832; doi:10.1038/s41598-020-60947-x)
Supplement: Supplementary file 1 — Supplementary Data. [file 41598_2020_60947_MOESM1_ESM.pdf]

Pancreatic islets seeded in a novel bioscaffold forms an organoid to rescue insulin production and reverse hyperglycemia in models of type 1 diabetes

Diana M. Elizondo[1], Nailah Z. D. Brandy[1], Ricardo L. L. da Silva[1,2], Tatiana R. de Moura[2], Jamel Ali[3], Dazhi Yang[1] and Michael W. Lipscomb[1]

[1]Department of Biology, Howard University, Washington, DC, United States

[2]Laboratório de Imunologia e Biologia Molecular-Hospital Universitário, Universidade Federal de Sergipe, Aracaju, Brazil

[3]Department of Chemical and Biomedical Engineering, FAMU-FSU College of Engineering, Tallahassee, Florida, USA, 32310
