# Supplementary figures and images for: Pancreatic islets seeded in a novel bioscaffold forms an organoid to rescue insulin production and reverse hyperglycemia in models of type 1 diabetes
Source: Sci Rep. 2020 Mar 9;10:4362. doi: 10.1038/s41598-020-60947-x (PMC7062832; doi:10.1038/s41598-020-60947-x)

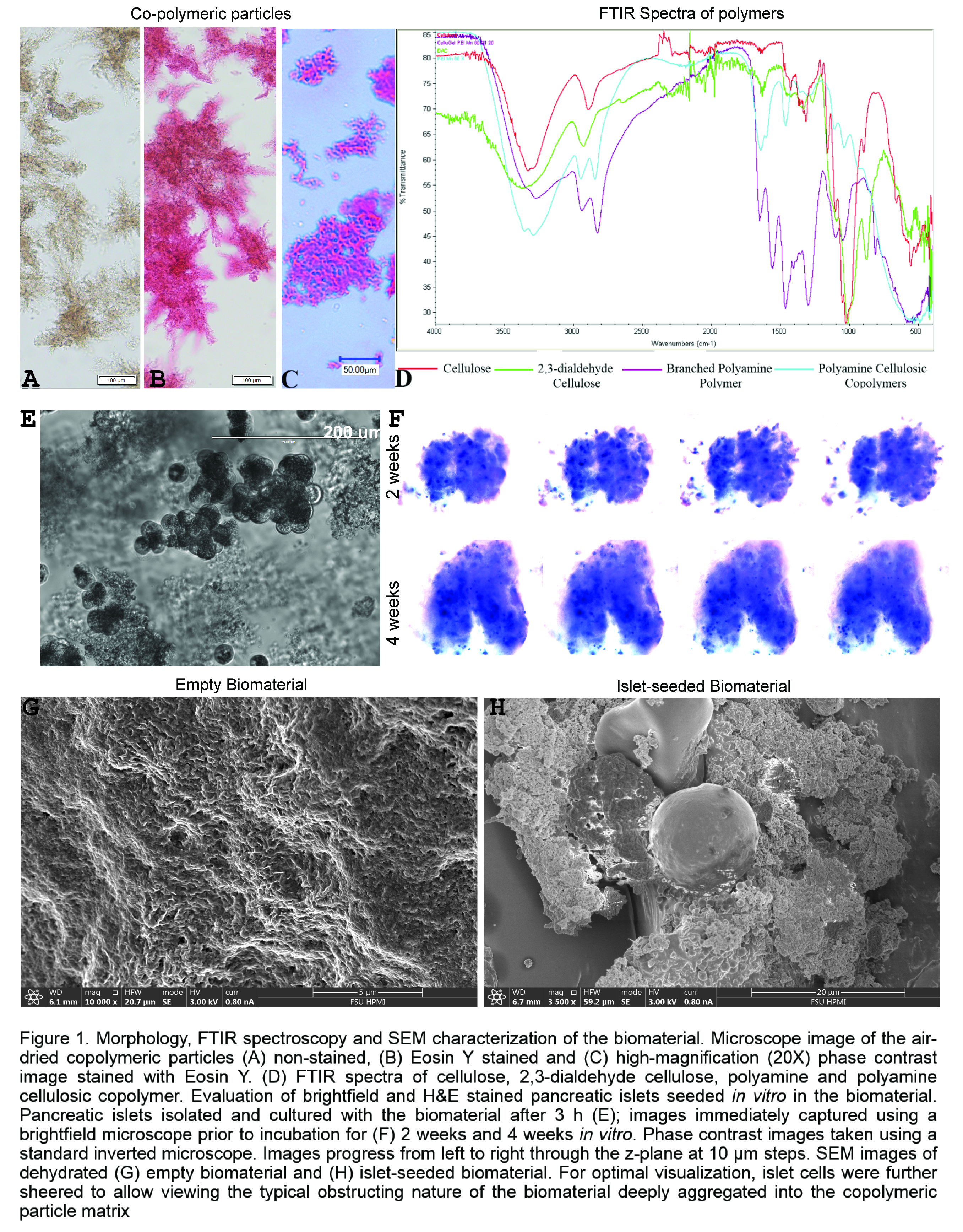

Supplement: Supplementary file 2 — Supplementary Data2. [file 41598_2020_60947_MOESM2_ESM.tif]

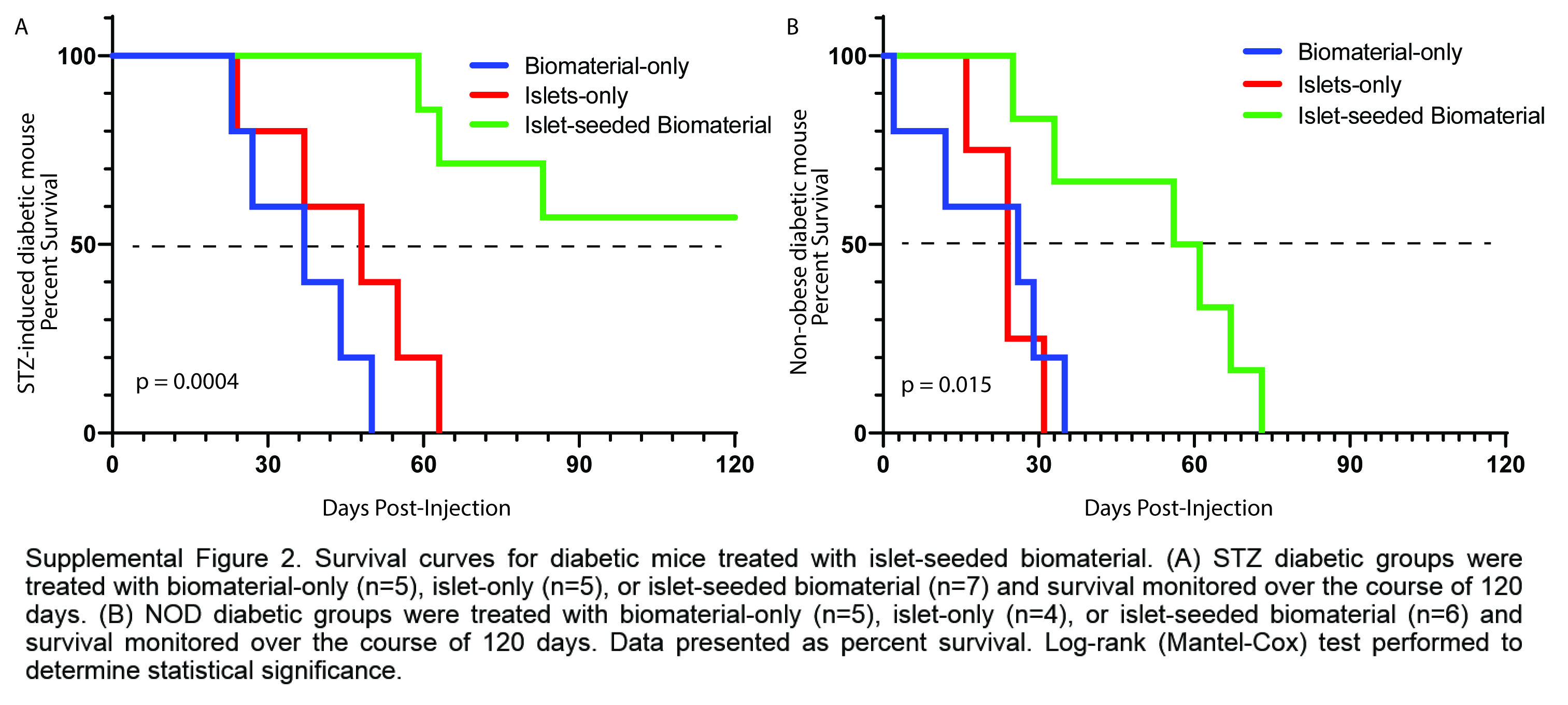

Supplement: Supplementary file 3 — Supplementary Data3. [file 41598_2020_60947_MOESM3_ESM.tif]

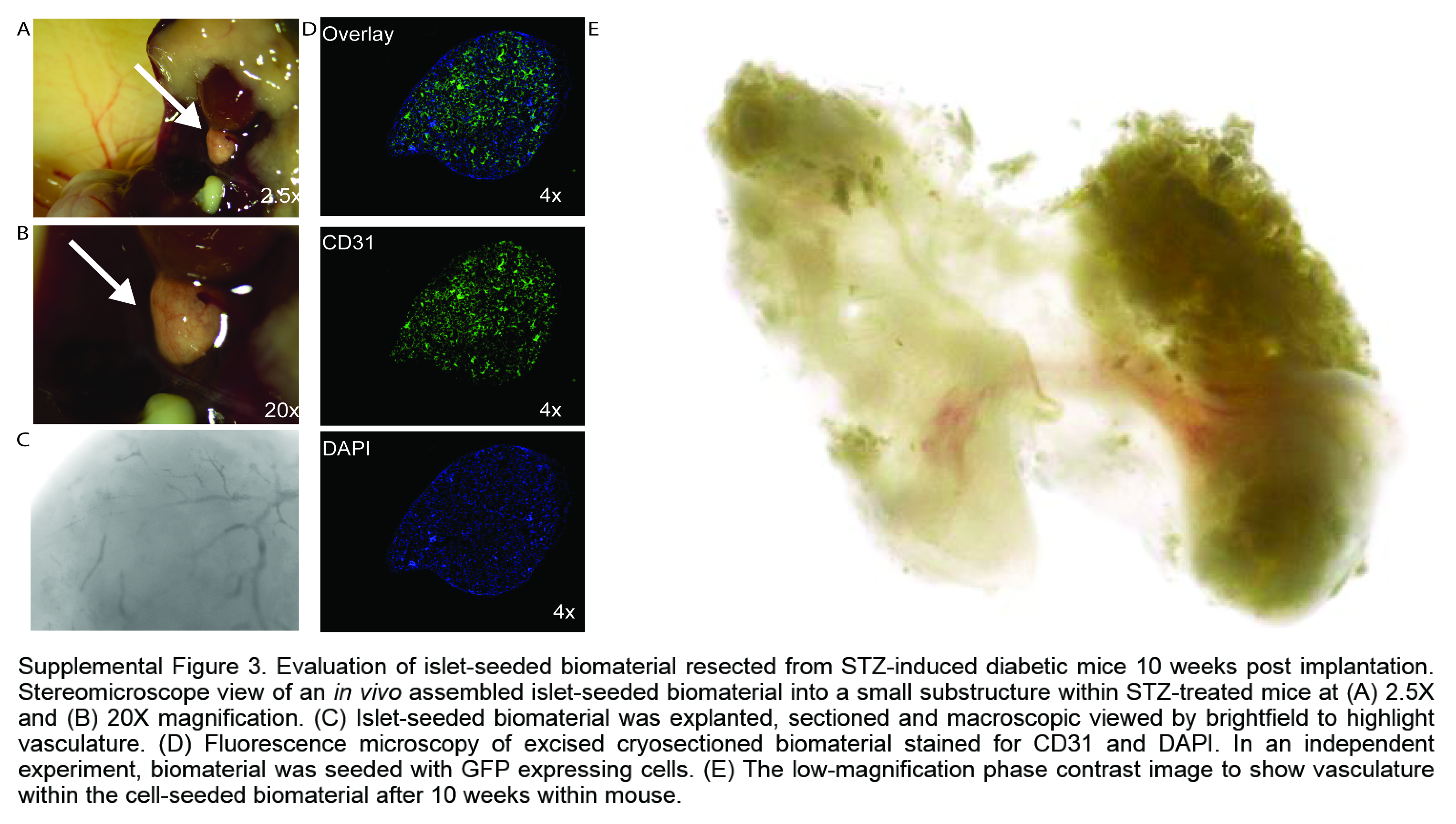

Supplement: Supplementary file 4 — Supplementary Data4. [file 41598_2020_60947_MOESM4_ESM.tif]
